# Occupational participation and engagement of woman experiencing premenstrual syndrome: A qualitative study

**DOI:** 10.1177/03080226231174792

**Published:** 2023-05-18

**Authors:** Yebin Park, Angela Murphy, Daniel Cezar da Cruz

**Affiliations:** School of Health, Leeds Beckett University, Leeds, UK

**Keywords:** Premenstrual syndrome, daily occupation, qualitative research

## Abstract

**Introduction:**

Premenstrual syndrome is commonly experienced by women of reproductive age and encompasses somatic, psychological and behavioural symptoms that recur during the luteal stage of menstrual cycle and resolves during or after menstruation. Although premenstrual syndrome has gained growing interest in recent years, the experience of women living with premenstrual syndrome has not yet been explored from an occupational perspective.

**Method:**

This qualitative study an used exploratory and descriptive design to gain a deeper understanding of the lived experience of women with premenstrual syndrome through an occupational lens. Data were collected using semi-structured interviews with four women living with premenstrual syndrome symptoms. Each interview was transcribed verbatim and thematically analysed.

**Findings:**

Three themes were identified: (1) occupational disturbance, (2) social impairment and occupational disengagement, and (3) the importance of self-awareness to engage in occupations. Women with premenstrual syndrome symptoms experienced difficulties that impacted their ability to engage in occupations including self-care, productivity and leisure, interfering with their occupational routine and interpersonal relationships. A level of awareness was considered important to manage premenstrual syndrome symptoms.

**Conclusion:**

The findings of this study are relevant to understanding the impact of premenstrual symptoms on daily living and for tailoring future interventions to address the occupational needs in women with premenstrual syndrome.

## Introduction

Premenstrual syndrome (PMS) is a combination of somatic, psychological and behavioural symptoms, which recurs during the luteal stage of the menstrual cycle and resolves during, or within several days after menstruation (Ismaili et al., 2016). The symptoms commonly experienced include depression, anxiety, fatigue, feeling bloated, general aches and pains, alteration in appetite and sleep disorders. According to Royal College of Obstetricians and Gynaecologists, the type or number of symptoms experienced may vary depending on the individual as psychological symptoms are predominant for some women while others may experience mixed symptoms (Green et al., 2017). Although little is known about the cause of PMS, mounting evidence points to a connection with heightened sensitivity to hormone levels associated with the menstrual cycle (Ryu and Kim, 2015). In addition, several factors such as age, aerobic exercise and nutrition have been identified to affect prevalence and/or intensity of PMS according to meta-analysis of data (Direkvand-Moghadam et al., 2014).

Approximately half of women of reproductive age worldwide experience PMS and about 10% of them have premenstrual dysphoric disorder (PMDD), which is a more severe form of PMS (Green et al., 2017). In spite of its high prevalence, PMS remains a largely under-researched issue, particularly in the field of occupational therapy. This study, therefore, purports to shed light on how PMS can impact individual’s daily life and quality of life through an occupational lens to lay the groundwork for developing evidence-based occupational therapy practice working with women experiencing PMS symptoms.

## Literature review

There is a body of literature investigating how the presence of PMS symptoms affects an individual’s daily life. In a study conducted by Choi et al. (2010) in South Korea, they have found that physical symptoms including back pain and abdominal discomfort are experienced more predominantly compared to psychological symptoms such as irritability and mood swings among participants and these have a strong correlation with an impairment on activities of daily living (ADL). This is supported by another population base study from Pakistan that reports the negative effect of the PMS symptoms on women’s ADL that correlate with the severity and duration of the disorder (Pal et al., 2011). These results were further confirmed in a larger-scale study conducted with women across 11 different countries, in which there was a high degree of similarities of PMS symptoms, regardless of where they lived, demonstrating a negative impact on their ADL (Dennerstein et al., 2011).

To date, a number of studies have demonstrated quality of life is decreased in female university students experiencing PMS (Rapkin and Winer, 2009; Sahin et al., 2014). With regard to the interactions between PMS symptoms and academic performance among the affected population, few studies have shown consistent results. A study conducted by Shehadeh and Hamdan-Mansour (2018) investigating female university students in Jordan found that the prevalence and severity of PMS negatively associated with academic involvement, self-determination and academic scores, while similar results were reported, more recently, among Turkish university students by Bilir et al. (2020). Additionally, Victor et al. (2019) demonstrated that the presence of PMS symptoms also associated with impaired social relationships among university students in Brazil as individuals tended to isolate and avoid social interaction with friends and families while experiencing PMS compared to symptom free weeks. Although in line with those performed in Iran (Farrokh-Eslamlou et al., 2015) and in Turkey (Goker et al., 2015) where PMS symptoms were shown to have a profound impact on the social relationships of university students compared to non-affected individuals, the other study found no significant association between the premenstrual symptoms and level of social participation and involvement among respondents (Shehadeh and Hamdan-Mansour, 2018). Though useful for determining how PMS symptoms correlate with a reduced or impaired ability to study and socially engage with others, there remains a lack of qualitative data to further explore their relationship, which may provide an in-depth understanding of individuals’ experiences and perspectives.

To determine the impact of PMS symptoms in the context of work, Heinemann et al. (2010) conducted an explorative study, in which 822 participants from four different nationalities (Austria, Brazil, Germany, and Spain) were instructed to complete the Premenstrual Symptoms Screening Tool and a modified version of the Work Productivity and Activity Impairment questionnaire based on the Daily Record of Severity of Problems questionnaire over 2 months. The result shows that there was a significant association between the severity of PMS symptoms and higher mean levels of absenteeism, which highlights a large economic burden that falls upon society through decreased productivity among the affected population. This is consistent with a more recent study by Hardy and Hunter (2021), which was conducted on female professionals (*n* = 125) in the United Kingdom. Their findings confirmed that PMS correlates with higher rates of work absence and highlighted the importance of presenteeism. However, the adverse effects on PMS on job performance were not observed, which is inconsistent with previous findings, therefore needs further investigation (Hardy and Hunter, 2021).

Indeed, literature has shown that PMS symptoms are associated with increased level of impairment in various aspects of occupations of an individual, consequently influencing their health and well-being. There is, however, a lack of detailed qualitative studies into lived experiences of those who are living with PMS symptoms (Direkvand-Moghadam et al., 2014). Moreover, to date, only one study has been published in occupational therapy literature that focuses on this area (Pekçetin et al., 2022). In their cross-sectional study, occupational performance and value of 35 university students experiencing PMDD were assessed using the Occupational Self-Assessment (OSA) and were compared with age-matched controls, in which the OSA competence score significantly differed between the two groups, while the OSA value score did not markedly differ (Pekçetin et al., 2022). In addition, it was further discovered that female students with PMDD symptoms find it more challenging to work toward goals and to concentrate on tasks at hand compared to those without the symptoms, which is in line with previous findings (Shehadeh and Hamdan-Mansour, 2018; Victor et al., 2019). Current knowledge about PMS symptoms from an occupational therapy perspective is greatly limited and requires further investigation. Moreover, the aim of this study is to fill the existing gap in the literature by providing a deeper insight into perceptions of women living with PMS symptoms, with focus on understanding how they affect their ability to participate in meaningful occupations.

## Method

This is a qualitative study using an exploratory and descriptive design, which is considered as an effective theoretical framework to examine topics within healthcare that previously gained little or no attention (Hunter et al., 2019). Three research questions were addressed: (1) what are the lived experiences of women with PMS? (2) To what extent does PMS influence their daily occupations? (3) What are the needs of women with PMS?

## Ethics

This study was approved by Leeds Beckett University’s ethics committee and was carried out as described in the approved protocol. A participants information sheet was provided via email, which contained the clear explanation of the nature and purpose of the study. Prior to participation, a written informed consent form was obtained from all the participants before starting any data collection. Interview questions were provided prior to the interview and participants were asked to let the researcher know of any questions concerning the study or its procedure. Participants were also provided with a debriefing sheet after participation, which contains information about helplines, crisis contacts, and other relevant organisations for self-help. Confidentiality was assured and pseudonyms were used in reporting the results.

## Participants

Participants were recruited through advertisements posted on relevant Facebook groups. Inclusion criteria were as follows: (1) female aged 18 or over; (2) experiencing PMS symptoms; and (3) able to speak, read and write in English. Those interested in participating contacted the researcher and were provided with the participant information sheet to explain the study in further detail. Two out of six women who provided written consent to take part, subsequently decided not to participate further due to time constraints and four completed interviews.

## Data collection

One-on-one semi-structured interviews were completed with four women who had been experiencing PMS symptoms. Each interview was arranged with participants via email and conducted using either Zoom or Microsoft Teams, depending on participants’ preferences. The interview comprised of 13 questions and probing was carried out, where appropriate, to encourage participants to elicit further responses (Sandelowski, 2010). The interview sessions ranged from 45 to 70 minutes in duration and were video recorded with prior consent of participants for transcription and data analysis. Identifying information was removed from transcripts to ensure anonymity and transcripts were stored securely on a password protected computer in accordance with ethical protocol and the General Data Protection Regulation.

## Data analysis

Data were analysed using inductive thematic analysis, based on the framework outlined by Braun and Clarke (2006). After each interview, transcripts were checked against the video recordings to increase accuracy, the researcher familiarised herself with the data through repeated reading, which was followed by organising and examining the raw data. A list of codes relating to each research question were developed and organised into categories to form sub-themes and three overarching themes. Subsequently, the emerged themes were further refined and named and were re-checked against the data for validity.

## Rigour

To ensure trustworthiness and authenticity, this study fulfilled several criteria outlined by Lincoln and Guba (1985). An audit trail linking all raw data, notes and codes with sub-themes and themes was ensured to ascertain credibility, while transferability was promoted through verbatim transcripts and rich and thick descriptions. Furthermore, reflective notes were taken during data collection and throughout the process of data analysis to enhance researcher reflexivity. The primary and second authors own experiences of PMS and influences on occupational participation and engagement were reflected upon and managed through careful consideration and reflection of possible influences on the research process. Assumptions and presuppositions were challenged in regular critical discussions involving the first and second authors throughout the research, identifying and managing influences on what was deemed important within the data. Care was taken to ensure the analysis was grounded in the participants own words and experiences. Researcher triangulation was considered by the three authors contributing with data analysis. The third author is a male and did not have influence by lived experience with the phenomenon involved in the research. The third author participated in the stages 5 and 6 (Braun and Clarke, 2006), refining the themes and final report. Theme 2 was modified from ‘Social Impairment’ to ‘Social impairment and occupational disengagement’ and theme 3 from ‘The importance of self-awareness’ to ‘The importance of self-awareness to engage in occupations’ to address the occupational perspective of the phenomenon (Njelesani et al., 2014). The three researchers met on MS Teams to discuss the changes and approve the final version of the article. These changes were important to differentiate occupational participation and engagement in participants lived experience (Cruz et al., 2023).

## Findings

Three main themes that describe the lived experiences of women living with PMS symptoms were identified in the interviews. These were (1) occupational disturbance, (2) social impairment and occupational disengagement, and (3) the importance of self-awareness to engage in occupations. Each of these themes is explored in detail below.

### Theme 1: Occupational disturbance

Each of the participants experienced a wide range of PMS symptoms and illustrated challenges or concerns regarding how such symptoms have interfered with their occupations. One participant Jane stated that she is less motivated to perform their daily routine occupations when experiencing PMS symptoms: ‘I do like everything nice and tidy in front. I do . . . But when I’m at that stage I don’t care’. Jennifer also shared a similar experience in regard with preparing meals:

I’ve got no energy to prepare any good food for myself. I just go with the easiest option. I actually enjoy cooking when I’m in a good place myself. But at that time, I have no energy and don’t want to bother. (Jennifer)

For Jennifer, decreased energy and motivation were described as a main barrier that hinders her ability to participate in work: ‘I didn’t care so much about work because I was just not feeling good in myself. So, I wasn’t able to give 100% . . .’, as well as in leisure activities: ‘I haven’t got the energy to do anything to make myself feel better. So, like going for a walk or just achieving little things like that is a massive task’. Other participants suggested that their work had been largely affected by ineffective emotional regulation during premenstrual cycle. As outlined by Carrie, ‘This morning, I couldn’t see my screen from crying . . . but also carrying on with my work’. This was exemplified by Lucy who worked as a teacher:

Sometimes I’d be teaching, and I’d come home and be like, ‘Oh my God, I want to teach for the rest of my life. It’s such an amazing job. Like I’m helping kids and I’m making a difference there’. And then the other two weeks of the month, I come home crying, ‘I can’t do this. I can’t handle the misbehaviours in the class, I can’t handle the disrespect’. I get so angry, and I get so triggered by it. (Lucy)

Alongside the impact on occupational participation, it was also found that the participants had experienced difficulties in maintaining occupational routine due to PMS symptoms. As outlined by Jane, ‘I would just get more lazier and harder to do things both mentally and physically’. A similar perspective was shared by Lucy: ‘Two weeks before period is horrendous . . . I can’t stick to a routine . . . And literally the week of my period, a couple of days into it, it’s like a cloud left. And I feel completely different’. As such, participants often described how their daily routine reflected whether they experienced the symptoms or not. Jennifer also explains:

I probably wouldn’t want to get up as early. Like, I’d want to sleep in or just lie on the sofa and not to do as much as I would do normally . . . my day wouldn’t be as structured as it usually is. (Jennifer)

The occupational disturbance can be understood as the impact of the PMS symptoms on a pattern of occupation (routines) for participants, affecting their motivation and mood to engage in a range of occupations such as cooking, working, walking, domestic activities and sleep patterns.

### Theme 2: Social impairment and occupational disengagement

The participants described how their social relationship with families and friends was negatively influenced by PMS symptoms. It was commonly illustrated by the participants how PMS symptoms may alter how they felt about themselves, as outlined by Jane: ‘I’m like a different person. I honestly thought I was bipolar at one point’. Similarly, Jennifer referred to herself as ‘Jekyll and Hyde’. Such changes undergone by the participants altered their ability to participate in social activities and the way they engaged with people they care about, as described by Lucy: ‘I tend to push them away. I don’t . . . I struggle to trust people’. Jane also illustrated, ‘My mind is racing. Yeah, it is. That’s why I alienate myself. I’d rather be by myself otherwise I will end up having a go at my boyfriend for no reason’. As such, participants reported that they would rather avoid social interaction when experiencing PMS symptoms, in an attempt to prevent from repeating relationship difficulties. Jennifer explained:

I prefer to be in a room by myself sometimes because it feels like a safe space. I can’t argue with anyone. I can’t get out and . . . take frustrations out on the people that you love and actually do genuinely care for you. You don’t know why you’re frustrated and why you take things out on people . . . you can’t explain it at the time. (Jennifer)

Some participants perceived that whether they were experiencing PMS symptoms or not was highly related to how satisfied they were with their relationship, which creates problems and relationship difficulties with their partner or spouse. Two of the participants’ descriptions were as follows:

One week I can be telling my husband our relationship is over because I’m so fed up with everything and he pisses me off and stuff . . . I’m not attracted to him and all this kind of stuff. Then I’ll have my period and I’ll be like ‘oh, I really love you now and everything’s okay’. (Lucy)

It definitely impacted me and my ex’s marriage. Obviously, there is a number of reasons why we separated, but my moods and things were one of them. Every time when it was a time of the month, we would end up in the cyclical arguments and that’s why our marriage ended. And it was like, I’d say, just like clockwork. (Carrie)

Self-blame and feeling guilty for having arguments with their loved ones were reported by some participants, as described by Jane: ‘It starts as a small argument and then escalates and becomes such a big thing. And then I feel so guilty afterwards’. Similarly, Jennifer echoed Jane’s concerns: ‘When I almost had a meltdown, I do feel terribly guilty because I can’t explain the reason for it. That’s just how I feel in my body at the time. It’s just pure frustration’. Preoccupied by PMS symptoms, some participants found it difficult to fulfil their occupational roles as a mother, wife, and a friend, which often led them to feel guilty, as outlined by Lucy: ‘I feel like I’m not being a good mom. I’m not being a good wife. I’m not being a good friend to people I like. What’s the point of me then?’. A similar account was reported by Carrie:

I want to be present for my family . . . I want to be playing with my son taking him out and doing stuff. But instead, I’m telling him ‘Yeah, you can have screens’. Because I don’t want him to see me crying. That’s no way to live. (Carrie)

The social impairment can be described as the consequences of PMS symptoms in the social environment (social groups) of participants and their relationships. The thoughts and feelings about themselves illustrated by frustration, anger and guilt affect the social participation since the strategy adopted by the respondents were to avoid social interaction and to remain isolated to cope with symptoms. Under an occupational perspective, participants need a time for disengagement of occupations to deal with their thoughts and feelings positively.

### Theme 3: The importance of self-awareness to engage in occupations

In terms of management of PMS symptoms, each participant placed importance on being aware of their symptoms and tracking their menstrual cycle, as outlined by Carrie: ‘It’s that awareness of it that just makes so much difference’. Jennifer also noted, ‘That’s really useful knowing when my cycle is because, unlike before, now I just know’. As such, awareness was considered as both ‘a good starting point’ (Jane) and a strategic plan to cope with psychological distress during the premenstrual phase. Lucy describes:

Now I can nip it in the bud when I start getting intrusive thoughts. Now I can be like . . . ‘you’re not real’, like, and I can distance myself from it a lot more effectively. (Lucy)

While personal strategies discussed by each participant differed from one another, all the participants highlighted the multifaceted nature of PMS symptoms sharing similar views on how healthy lifestyle choices and behaviours must be accompanied by increased efforts to reduce their symptoms. Two of the participants’ descriptions were as follows:

I know that if I take the right supplements, if I eat the right foods, if I do the meditation, the yoga and the exercise and the journaling, my symptoms are not quite as bad. And I know that I cannot eliminate them but can reduce them (Carrie):

After six months of counselling and therapy and antidepressants, I realised that I basically have to treat myself. And by improving my diet, by improving my exercise, by meditating, I was reading about Buddhism a lot to kind of get the psychological things in perspective a lot more. And those things really, really helped me. (Lucy)

As such, a number of occupations such as doing yoga, taking a walk, going for a run as well as meditating and journaling were frequently discussed by the participants and identified as of importance in efforts to de-escalate premenstrual symptoms. The participants, however, complained of their difficulties implementing this into their daily routine, as Carrie described:

It’s more of a struggle to do them. I took myself to yoga this morning, I didn’t want to go, so I kind of forced myself . . . I did feel better for it, but I did just want to curl up in a little ball. (Carrie)

Similarly, Jennifer explains:

I’m so bad at trying to push myself because I’m constantly in the battle against myself. But there have been times where I have taken myself on a walk and I thought ‘yeah . . . that has made me feel better’. But I have to really push myself to do it. (Jennifer)

The importance of self-awareness described as one of the first steps taken by participants to alleviate or reduce the symptoms of PMS. Although they reported the traditional pharmacological treatment as a possibility, they explored their occupational nature of improving their self-awareness with regard to what type of occupations makes them feel better. Despite this knowledge, they seem to struggle with their routines to allow them to regularly engage in occupations that would lead them to well-being.

## Discussion and implications

The findings of this study have suggested that PMS symptoms pose a barrier to occupational performance and engagement in areas of self-care, productivity and leisure. Symptoms experienced significantly varied, so did the way they interfered with participants’ daily occupational participation and engagement. It was identified that feeling fatigued and low energy were closely associated with psycho-behavioural outcomes such as decreased motivation and lack of interest in occupational participation and engagement, regardless of how important or meaningful the occupations may be. This finding is consistent with a previous investigation by Tolossa and Bekele (2014), where female university students in Ethiopia (*n* = 258) answered survey questions regarding prevalence, impact and management of PMS and over three-quarter of participants (77.5%) reported having experienced loss of interest in engaging in occupations during premenstrual weeks. All of the participants also spoke about how PMS symptoms had an impact on work participation and other productivity occupations. In line with the previous findings of Matsumoto et al. (2013), who found a strong relationship between PMS and higher absenteeism and decreased work productivity, participants reported that they have had reduced working hours or changed jobs because of difficulties associated with PMS symptoms including impaired concentration, irritability and low morale. This may be associated with the fact that only one participant was currently employed even though all of them were of working age, though this requires investigation in future studies.

Another important finding involves difficulties maintaining occupational routines during the premenstrual phase. This is not entirely surprising, given that PMS symptoms have been shown to interfere with all areas of daily occupations. In social psychology, having a structured routine on a daily basis has been said to act as an anchor for an individual, particularly when in distress; and in turn, provide a sense of security, confidence and meaningfulness in life (Avni-Babad, 2011; Mohideen and Heintzelman, 2022). Indeed, the concept of routine is well known in occupational therapy literature. Not only has this been placed as the core principle of the discipline in the course of history (Epley et al., 2021), it is also tightly linked with the development of theoretical frameworks within the profession, such as the Model of Human Occupation (MOHO) (Kielhofner and Burke,1980; Kielhofner and Forsyth, 1997). In MOHO, routines are part of habituation and comprise the habits of performance, style and routine that people fulfil when they engage in their occupational roles and that becomes a pattern when they regularly perform (Taylor, 2017). In this sense, as the examples presented in our findings, if the motivation affects occupational participation, then routines can be disrupted.

As such, having an occupational routine is perceived and valued to facilitate both occupational performance and optimisation of energy consumption throughout the day and has shown its effectiveness in enhancing the care of clients with mental health difficulties (Doroud et al., 2015) as well as the management of chronic diseases such as diabetes (Thompson, 2014). Therefore, occupational therapists may use their unique knowledge and skills to support the needs of women experiencing PMS.

According to the study conducted by Schmelzer et al. (2015), women with PMS symptoms are significantly more likely to experience the impairment in the social domain. This was reflected in the findings of the current study where participants reported negative outcomes in social interaction and engagement with others within their environment. The participants felt that PMS symptoms had a detrimental effect on their characteristics, thoughts and beliefs, resulting in the loss of sense of self, which was manifested as feelings of frustration, anger and hopelessness towards themselves as well as other people around them. The inability to effectively regulate such emotions was described as one of the main reasons why the participants had difficulties in engaging in social interaction and was related to the intensity and frequency of interpersonal conflicts, particularly with partners, which echoes the findings from Karimiankakolaki et al. (2019). It was also revealed that such patterns of difficulties in interpersonal relationships were often followed by feelings of guilt and self-blame. As a result, participants illustrated being more occupationally disengaged and isolated to prevent themselves from repeating past mistakes. This finding supports and builds upon earlier research by Victor et al. (2019), who established the association between social avoidance and PMS, with an insight into the reasons underlying such phenomenon. Social impairment among affected populations not only may lead to the absence or lack of support from families and friends, which has been recognised as a key factor in premenstrual coping (Eissa, 2010), but also could cause occupational loss in social activities, which, in turn, further influences negative health outcomes and one of the reasons is the disconnection with others, belonging (Hammell and Iwama, 2012).

Participants also shared similar views on awareness of their PMS symptoms and how it allowed them to develop personalised strategies to cope with difficulties more efficiently. This finding confirms that of Ussher and Perz (2013), who suggested that women benefit from accepting and being aware of premenstrual changes in that they are more inclined to participate in other behavioural coping strategies. Moreover, the findings also note that occupational engagement in meaningful occupations has been perceived as a helpful and effective way among participants that had made them feel better and alleviated perceived PMS symptoms, yet was often disrupted due to premenstrual distress, feelings of laziness or lack of motivation. This implies that the advantages and necessity of pursuing occupations that are meaningful and purposeful may be overlooked and undervalued by women with PMS symptoms; however, future investigations are needed to verify this point. Occupational therapy has the potential to contribute to people experiencing PMS symptoms to maximise their self-management with occupation-centred interventions (focused and based). Occupational participation and engagement can be facilitated through a routine plan and exploring the power of occupations to alleviate PMS symptoms, enabling positive occupational engagement (Morris and Cox, 2017).

## Limitations

One of the limitations of this study includes the small number of participants. This was because only six women meeting the inclusion criteria showed an interest and agreed to take part. Two of them withdrew from the study due to limitations in time and interest. Though the sample size in qualitative studies can be justifiable (Boddy, 2016), this implies that theoretical data saturation may have not been achieved. Thus, some caution is needed when interpreting the results of this study. However, considering the originality of the theme and the lack of studies within the occupational therapy and occupational science, we believe the data presented consists of valuable evidence to demonstrate how PMS symptoms can impact on participants occupations, demonstrating that there is a potential contribution of occupational therapy to those experiencing difficulties with their routines and to self-manage PMS symptoms to enable them to engage in occupations. Another limitation is that member checking was not possible due to constraints of time to contact participants; however, the study addressed rigour by adopting researcher triangulation, tick description, and reflexivity (Curtin and Fossey, 2007).

Also, further questions to capture personal characteristics such as cultural background, marital status and socio-economic status could have been employed to analyse their influence on women’s experiences related to premenstrual symptoms. Another limitation is related to data on comorbidities. Two of the participants have briefly spoken about having psychiatric comorbidities during the interviews, but precise information was, unfortunately, not collected as a part of the present study. Therefore, future studies may benefit from incorporating a larger sample size taking into consideration factors associated PMS such as the prevalence of comorbidities to provide more comprehensive data.

## Implications for occupational therapy

Despite the high prevalence rates and many negative consequences, PMS remains poorly understood and described in existing literature. The findings of the current study have provided an initial step towards a better understanding of how PMS symptoms affect daily occupations by offering more insight into individuals’ lived experiences and built upon that of Pekçetin et al. (2022), who preliminarily addressed this underexplored issue in occupational therapy literature. Upon taking into consideration the multifaceted nature of premenstrual symptoms, the use of collaborative and client-centred approaches should be ensured by health professionals including occupational therapists when working with women with PMS. Furthermore, unique knowledge and skills from occupational therapy may give rise to a more comprehensive understanding of how to address the unmet needs of the affected population.

## Conclusion

This study has added to the limited evidence base of what is known about the lived experiences of women living with PMS by utilising in-depth qualitative methodology. The findings have provided deeper insights as to how PMS symptoms are experienced by individuals and the way they adversely affected their abilities to participate and engage in occupations, consequently impacting on the occupational routines of the affected population. Additionally, social impairment among the women with PMS has been highlighted and was found to be associated with psycho-behavioural consequences of premenstrual symptoms according to the findings. As suggested previously, self-awareness does play an important role in determining coping strategies in relation to premenstrual symptoms. The study’s findings also indicate that occupational therapists have potential to provide support in developing individualised strategies for PMS rehabilitation and management.

Despite the limitations of the current study, several contributions have been made to existing literature and the results further highlight the need to build more in-depth evidence on women living with PMS. Therefore, future research will be directed towards further expansion of understanding of impact on occupational performance and engagement associated with premenstrual symptoms by employing a larger and more diverse sample to further contribute to evidence needed to develop the role of occupational therapy practice for women experiencing PMS.

**Key findings**

- Women with PMS had difficulties participating in all areas of occupations, leading to challenges in maintaining occupational routine and engagement.
- Psycho-behavioural symptoms related to PMS may influence an individual’s ability to participate socially and occupationally.

**What the study has added**

This study highlighted the need to build more in-depth evidence on women living with PMS.
